# Emotion regulation, physiological arousal and PTSD symptoms in trauma-exposed individuals

**DOI:** 10.1016/j.jbtep.2014.03.002

**Published:** 2014-09

**Authors:** Laura Shepherd, Jennifer Wild

**Affiliations:** a Department of Clinical Psychology & Neuropsychology, Nottingham University Hospitals NHS Trust, UK; b Department of Experimental Psychology, University of Oxford, South Parks Road, Oxford, England OX1 3UD, UK

**Keywords:** Emotion regulation, PTSD, Trauma, Skin conductance, Reappraisal, Suppression

## Abstract

**Objectives:**

Retrospective studies suggest a link between PTSD and difficulty regulating negative emotions. This study investigated the relationship between PTSD symptoms and the ability to regulate negative emotions in real-time using a computerised task to assess emotion regulation.

**Method:**

Trauma-exposed ambulance workers (*N* = 45) completed self-report measures of trauma exposure, PTSD symptoms and depression. Participants then completed a computer task requiring them to enhance, decrease or maintain their negative emotions in response to unpleasant images. Skin conductance responses (SCR) were recorded and participants also made ratings of emotion intensity. Immediately after the computer task, participants were asked to describe the strategies they had used to regulate their negative emotions during the task and recorded spontaneous intrusions for the unpleasant images they had seen throughout the following week.

**Results:**

PTSD symptoms were associated with difficulty regulating (specifically, enhancing) negative emotions, greater use of response modulation (i.e., suppression) and less use of cognitive change (i.e., reappraisal) strategies to down-regulate their negative emotions during the task. More intrusions developed in participants who had greater reductions in physiological arousal whilst decreasing their negative emotions.

**Limitations:**

PTSD was measured by self-report rather than by a clinician administered interview. The results suggest a relationship between emotion regulation ability and PTSD symptoms rather than emotion regulation and PTSD.

**Conclusions:**

Difficulty regulating negative emotions may be a feature of trauma-exposed individuals with PTSD symptoms, which may be linked to the types of strategies they employ to regulate negative emotions.

## Highlights

• Emotion regulation can be measured in real-time in trauma populations.
• Skin conductance response provides a physiological measure of emotion regulation.
• PTSD symptoms were associated with difficulty enhancing negative emotions.
• Greater PTSD symptom severity was linked to greater use of suppression and less use of cognitive reappraisal.
• Reduced arousal during emotion regulation was related to subsequent intrusions.

## Introduction

Difficulty regulating negative emotions has been linked to the onset and maintenance of anxiety and depression (Campbell-Sills & Barlow, 2007; Gross, 1998a). More recent studies also suggest a relationship between emotion regulation difficulties and post-traumatic stress disorder (PTSD; Bonn-Miller, Vujanovic, Boden, & Gross, 2011; Cloitre, Miranda, Stovall-McClough, & Han, 2005; Eftekhari, Zoellner, & Vigil, 2009; Ehring & Quack, 2010; Kashdan, Uswatte, & Steger, 2006; Moore, Zoellner, & Mollenholt, 2008; Price, Monson, Callahan, & Rodriguez, 2006; Tull, Barrett, McMillan, & Roemer, 2007; Vujanovic, Bonn-Miller, Potter, Marshall, & Zvolensky, 2011). Whilst promising, these studies are limited to retrospective designs and reliant on self-report questionnaires to assess emotion regulation skills, including strategies typically used, as well as self-reports of emotion intensity. The difficulty with these designs is that they rely on participants accurately reporting and being aware of the intensity of their emotions and how they regulate them. Since discrepancies between self-report and physiological measures of emotion intensity have been found, the assessment of emotion regulation should ideally incorporate both self-report and objective (i.e. physiological) measures. This is especially important because deficits in emotion regulation can manifest as chronically elevated subjective negative affect relative to physiological activity; this is regardless of the level of environmental demands (Connelly & Denney, 2007). It is unclear whether PTSD symptom severity is linked to objective (physiological) difficulties in regulating negative emotions as opposed to perceived difficulty regulating emotion. The current study aimed to address this gap in the literature.

Gross (1998a) proposed a process model of emotion regulation, linking the timing of emotion regulation strategies to their effectiveness. The strategies used early in the process of generating an emotion are known as antecedent-focused and are thought to be more effective than those employed once an emotion is already underway, known as response-focused strategies (Gross, 1998a; Gross & Thompson, 2007). The model outlines three categories of strategies individuals may use to regulate their emotions, both positive and negative, once in any given situation. Attentional deployment is the first and refers to strategies to direct attention, such as choosing to focus on a particular part of the situation or environment. The second category is cognitive change and includes strategies, such as cognitive reappraisal, which alter the meaning of a situation to change its emotional impact. Response modulation is the third category and includes response-focused strategies, such as the suppression of emotion or expressive suppression (the latter of which refers to the suppression of observable indicators of emotion, such as suppressing facial expressions) or drug use, to influence physiological, experiential or behavioural reactions.

PTSD has been associated with emotion regulation strategies involving response modulation such as emotion suppression and expressive suppression whereas cognitive change (reappraisal) is generally reported to be under-utilised (e.g. Eftekhari et al., 2009; Ehlers & Clark, 2000; Ehring & Quack, 2010; Feeny & Foa, 2005; Marx & Sloan, 2005; Moore et al., 2008). More recently, Boden et al. (2013) prospectively investigated the relationship between emotion regulation strategies at intake for residential group CBT for PTSD and PTSD symptom severity at discharge in a sample of military veterans. Expressive suppression was associated with greater PTSD symptom severity whereas cognitive reappraisal was associated with fewer PTSD symptoms. Additionally, change in the use of expressive suppression during treatment predicted PTSD symptom severity at discharge even after accounting for baseline PTSD symptom severity. The greater the decrease in an individual's use of expressive suppression during treatment, the lower the PTSD symptom severity scores at discharge. These results further highlight the tendency for those with greater PTSD symptoms to use response modulation strategies more often and cognitive change strategies less frequently.

While the aforementioned studies suggest a link between self-reported difficulties in emotion regulation and PTSD in groups of individuals exposed to a range of trauma, including women who have experienced childhood sexual abuse (Cloitre et al., 2005), military veterans (Boden et al., 2013; Kashdan et al., 2006) and other trauma-exposed populations (Bonn-Miller et al., 2011; Eftekhari et al., 2009; Ehring & Quack, 2010; Tull et al., 2007), they all relied on self-report measures of emotion regulation incorporating a retrospective design. Only one study could be found which assessed the ability of trauma-exposed individuals to regulate negative emotions in real-time. Following the aftermath of the 9/11 terrorist attacks, Bonanno, Papa, Lalande, Westphal, and Coifman (2004) presented New York college students with unpleasant images on a computer screen. Participants were instructed to enhance or decrease their negative emotional responses to the images. Those who were better able to enhance and decrease their negative emotions showed less psychological distress by the end of the second year following these attacks. Although PTSD symptomatology was not measured, this study provides preliminary evidence that difficulty regulating negative emotions on-line can be measured using experimental tasks and is linked to the development of psychological distress following trauma. However, as in the other studies, these authors relied only on self-report ratings of the intensity of emotional experience during the experimental task and a physiological measure of emotion regulation was not adopted.

A possible way forward in this important area of research would be to incorporate an objective physiological measure when assessing the ability to regulate negative emotions. One study has used concurrent assessment of physiology in addition to subjective reports of emotional experience in a healthy student population using an experimental task. Jackson, Malmstadt, Larson, and Davidson (2000) presented students with unpleasant and neutral images on a computer screen with instructions to enhance, decrease, or maintain their emotional responses whilst startle eyeblinks were measured. Instructions to decrease negative emotions led to smaller startle eyeblinks and instructions to enhance negative emotion led to larger startle eyeblinks. This was an important study in suggesting that an experimental task could be used to measure emotion regulation objectively through the assessment of its effects on physiological activity.

Since measuring skin conductance responses (SCR) is a less intrusive mode of measuring physiological arousal compared to startle eyeblinks, we chose SCR as a physiological measure of emotion regulation for our trauma-exposed participants. To our knowledge, no study has yet investigated the regulation of negative emotions and the corresponding effect on SCR in trauma-exposed participants without any training in emotion regulation being provided. We measured SCR and self-report ratings of emotion as indicators of emotion regulation ability whilst trauma-exposed participants were instructed to enhance, maintain or decrease their negative emotional responses to unpleasant images presented during a computer task. Participants were not provided with instructions or training regarding how they might regulate their negative emotions. Participants were also asked to record intrusions related to the computer task for the week following participation using diaries since previous research has suggested that reductions in physiological arousal whilst being shown unpleasant images in the form of traumatic films may lead to the development of trauma-related intrusive memories (e.g., Holmes, Brewin, & Hennessy, 2004).

Our aims were fourfold: (1) to validate the experimental task and the use of SCR to measure the regulation of negative emotions in real-time in trauma-exposed individuals who had not been provided with any specific instructions or training, (2) to explore whether emotion regulation was related to PTSD symptom severity, (3) to investigate whether specific strategies (cognitive change and response modulation) were linked to greater PTSD symptom severity, and (4) to assess the relationship between changes in arousal during attempts to regulate negative emotions and the subsequent development of intrusive memories.

In relation to our first aim, it was predicted that self-reported and objective (i.e., SCR values) indices of emotion regulation would be greater towards unpleasant images compared to neutral images, and would be greatest following instructions to enhance, smallest following instructions to decrease, and in between following instructions to maintain initial negative emotional responses towards unpleasant images. We also hypothesised that difficulty regulating negative emotions on the computer task would be associated with greater PTSD symptom severity. Due to the novel nature of the design, we made no assumptions or hypotheses about whether emotion regulation difficulties in those with greater PTSD symptoms would be present in all or in just some conditions (i.e., following instructions to enhance, maintain or decrease negative emotions). Since healthy participants are able to enhance and decrease negative emotions in response to negative pictures with corresponding effects on physiology (i.e., Kim & Hamann, 2012; Ray, Ochsner, McRae, & Gross, 2010), difficulty regulating emotion in response to any of the instructions would be suggestive of emotion regulation difficulty. It was therefore hypothesised that PTSD symptom severity would be related to difficulty enhancing, decreasing or maintaining initial negative emotions in response to unpleasant images. It was further hypothesised that PTSD symptom severity would be associated with greater use of response modulation and less use of cognitive change strategies. Finally, we predicted that decreases in arousal during emotion regulation attempts would be associated with an increased frequency of intrusive memories for the unpleasant images presented during the computer task over the following week.

## Method

### Participants

Forty-five ambulance workers (31 male) were recruited. The mean age of participants was 37 years (range: 26–60 years; SD = 8.7 years). Forty-four of the participants were White British and one was British Asian. Sixty-two percent (*n* = 28) of participants were single, 24% (*n* = 11) were married, 11% (*n* = 5) were divorced, and one participant preferred not to specify. On average, participants had been working as ambulance workers for 8.6 years (range: 10 months–30 years). Psychophysiological recordings were measured using the non-dominant hand. In the sample, 87% (*n* = 39) of participants were right-handed.

### Materials

#### Self-report questionnaires

Previous exposure to traumatic events was measured by a modified version of the trauma list in the Clinician Administered PTSD Scale (CAPS; Blake et al., 1995). Symptoms of PTSD were measured with the Post-Traumatic Stress Diagnostic Scale (PDS; Foa, Cashman, Jaycox, & Perry, 1997). The PDS is a widely used measure of PTSD symptoms with 17 items. Possible scores range from 0 to 51. Scores of ≤10 are classified as ‘Mild’, between ≥11 and ≤20 as ‘Moderate’, between ≥21 and ≤35 as ‘Moderate to Severe’ and ≥36 as ‘Severe’. Symptoms of depression were measured with the Beck Depression Inventory (BDI; Beck, Rush, Shaw, & Emery, 1979), a reliable and valid 21-item measure of depressed mood. Possible scores range from 0 to 63. Scores of 0–9 indicate that a person is not depressed, 10–18 indicates mild-moderate depression, 19–29 indicates moderate-severe depression and scores over 30 signify severe depression. Emotion intensity was measured with a Likert scale ranging from 1 to 9 (weak to strong). After responding to an emotion regulation instruction, participants were asked to rate the strength of their emotion linked to each image on this scale. To assess the emotion regulation strategies participants employed during the task, we administered the Self-Reported Emotion Regulation Strategies Questionnaire (adapted from Jackson et al., 2000). This questionnaire asked two open-ended questions: “What strategy or strategies did you use to suppress your negative emotions?” and “What strategy or strategies did you use to enhance your negative emotions?” Participants were required to explain in their own words what strategy or strategies they had used for each condition. Participants were permitted to write down as many strategies as they believed they had used during the computer task. For each strategy participants described, they were prompted to estimate the percentage of time they had used that strategy (from 0 to 100% of the trials) during the computer task. In the week following the computerised task, participants were asked to record daily how often they had experienced spontaneously occurring intrusive memories of any of the unpleasant images that were presented to them during the computer task in an intrusive memory diary.

#### Study stimuli

Thirty unpleasant pictures (valence < 3; arousal > 6) and 10 neutral pictures (valence 4.5–5.5; arousal < 3) were selected from the International Affective Picture Set (IAPS; Center for the Study of Emotion and Attention (CSEA-NIMH, 1995). Unpleasant images included various types of bodily injury, death and violence. Ten neutral images were selected and included common household or other objects. The 40 images (30 unpleasant, 10 neutral) randomly appeared on a computer screen for 12 s in total. The methodology was adapted from Jackson et al. (2000). Each trial followed five steps: 1) a randomly selected image appeared on the computer screen and participants were instructed to simply observe the image for 4 s. This served as the initial emotional response period; 2) at 4 s post-image onset, a digitised human voice gave participants a one-word emotion regulation instruction whilst the image remained on the computer screen. For unpleasant images, participants were randomly instructed to ‘ENHANCE’, ‘MAINTAIN’ or ‘DECREASE’ their emotional response. For neutral images, they were only ever instructed to ‘MAINTAIN’ their emotional response; 3) for the remaining 8 s of the image presentation, participants attempted to regulate their emotional responses as instructed; 4) the image then disappeared from the computer screen and a Likert scale appeared. Participants were instructed to rate the strength of their negative emotion on a scale of 1–9 (weak to strong); 5) after making their rating, the word ‘RELAX’ appeared on the screen for 10 s, after which the next image appeared.

#### SCR monitoring

SCR was measured throughout. Individual baseline physiological arousal was controlled for. This was done by recording SCR for around five to 10 min prior to starting the task to ensure that participants' physiological arousal had stabilised and presumed to reflect a relatively relaxed state. This SCR was set as a baseline for each participant and the task was started. This meant that participants' SCR in response to stimuli presented during the task was comparative to each participants' baseline physiological arousal and controlled for individual differences in baseline physiological arousal. PowerLab 4/30 data acquisition system with LabChart Pro was used to measure SCR during the computer task. LabChart Pro software extracted the data for analysis.

### Procedure

Participants completed the self-report measures of trauma exposure, PTSD symptoms and depression. They were then prepared for SCR recording following standard procedures. Two SCR electrodes were attached to the middle phalanges of the first and third fingers of the non-dominant hand. After an individual baseline SCR was established, SCR recording began and participants completed the computer task (e.g., Dawson, Schell, & Filion, 2000).

Using standard procedure, the minimum response amplitude detected as a valid SCR was 0.05 μS, and the SCR response was measured 1–4 s post-stimulus onset to gain a measure of an initial emotional ‘response’ to each image (e.g., Dawson et al., 2000). This ensured that non-specific arousal or arousal unlikely to be produced by the image was not measured. SCR was then measured 1–4 s post-instruction and served as the emotion ‘regulation’ phase. Thus, following standard procedure, 1–4 s post-stimulus onset was taken to reflect initial response to the stimulus. At this point, participants then heard an instruction to ‘enhance,’ ‘maintain’ or ‘decrease’ their emotions. A timeframe of 1–4 s post-instruction was taken to reflect the emotion regulation period. Responses generated more quickly than 1 s post-instruction and following 4 s post-instruction were considered to be non-specific activity and thus not included. The rate of the SCR response was averaged across the above timeframes and across computer task condition. The rate of the SCR response was analysed given that this provides a measure of the change in the SCR response over time, which was the focus of the study. As detailed in the prior section, individual participant variation in baseline physiological arousal was controlled for.

Following the computer task, participants then recorded the strategies they had used to regulate their emotions. Finally, they kept a diary to record any intrusive memories related to the unpleasant images presented during the computer task for the following seven days.

### Data analysis

Variables violating the assumption of normality (age, intrusions following the computer task, PDS scores and all SCR data) were log transformed. Examination of the skewness and kurtosis values following log transformation indicated that this was successful.

To validate the computer task methodology, repeated measures *t*-tests and ANOVA analyses were conducted to test the hypotheses that self-reported and objective (i.e. SCR values) indices of emotion regulation would be greater towards unpleasant images compared to neutral images, and would be greatest following instructions to enhance, smallest following instructions to decrease, and in between following instructions to maintain initial negative emotional responses towards unpleasant images. To examine the hypothesis that difficulty regulating negative emotions on the computer task would be associated with greater PTSD symptom severity, Pearson correlation analyses were conducted. We also predicted that PTSD symptom severity would be associated with greater use of response modulation and less use of cognitive change strategies. Pearson correlation analyses were conducted to test this hypothesis. Finally, we conducted Pearson correlation analyses to test the hypothesis that decreases in arousal during emotion regulation attempts would be associated with an increased frequency of intrusive memories for the unpleasant images presented during the computer task in the subsequent week.

Pairwise comparisons were conducted when appropriate and were adjusted for multiple testing using Bonferroni values. Bonferroni adjusted *p* values were also applied to correlational analyses. Effect sizes (Cohen, 1992) were calculated for every finding as advised by the American Psychological Association (1994) since these express the importance of the research findings without relying on whether the test performed was one- or two-tailed or on *p* values. Cohen (1992) proposed a threefold effect size classification system for *d* values: small (*d* = 0.20–0.49), medium (*d* = 0.50–0.79), and large (*d* = 0.80 and above), and a threefold classification system for *r* values: small (*r* = 0.10–0.29) medium (*r* = 0.30–0.49) and large (*r* = 0.50 and above) and for *F* values: small (*F* = 0.10–0.24), medium (*F* = 0.25–0.39) and large (*F* = 0.40 and above). SCR data were lost for two participants due to technical failure; therefore, analyses including SCR data are based on 43 participants.

## Results

### Participant sample

Sixty-seven percent (*N* = 30) of the sample were classified as suffering from a mild level of PTSD, 26% (*N* = 12) from a moderate level of PTSD and 7% (*N* = 3) from a moderate-severe level of PTSD. The mean total score on the PDS was 7.4 (SD = 7.6, range 0–28), falling in the mild range of severity. Male and female participants' mean scores on the PDS were not significantly different, *t*(43) = −0.18, *p* = .86, two-tailed. PTSD symptoms were not significantly correlated with years working for the ambulance service (*r* = 0.13, *N* = 45, *p* = .41, two-tailed). The mean BDI score was 8.5 (SD = 7.7, range 0–25), falling in the non-clinical range.

The mean number of different types of traumatic events previously experienced or witnessed by participants was 9.4 (range: 2–15; SD = 3.3). The most commonly reported traumatic events were witnessing others die/being seriously hurt (100% of participants), serious traffic accident (93%), and witnessing or coming across a suicide (91%). Although the PTSD scores for participants most commonly fell in the mild range, the sample had been exposed to a significant number and different types of traumatic events during their working and personal lives. The frequency of exposure to different types of trauma did not differ between male and female participants, *t*(43) = 1.89, *p* = .07. There was a statistically significant relationship between the frequency of previous exposure to different types of trauma and current PTSD symptoms, *r* = 0.39, *N* = 45, *p* < .01, two-tailed.

### Validating the methodology

#### Initial responses (1–4 s post-stimulus onset)

As predicted, the mean self-report rating of emotional response towards unpleasant images (*M* = 4.68, SD = 1.77) was significantly higher than the mean rating towards neutral images (*M* = 1.38, SD = 1.1, *t* = 17.24, df = 44, *p* < .001, one-tailed; Cohen's *d* = 2.23). As predicted, there was also a greater SCR rate of response towards unpleasant images (*M* = 0.05, SD = 0.1) compared to neutral images (*M* = 0.02, SD = 0.11, *t* = 1.89, df = 42, *p* = .03, one-tailed; Cohen's *d* = 0.29).

### Emotion regulation (1–4 s post-instruction)

Self-report ratings were smallest following instructions to enhance (*M* = 5.14, SD = 1.96), greatest following instructions to decrease (*M* = 4.25, SD = 1.74) and in-between following instructions to maintain (*M* = 4.65, SD = 1.85) negative emotional responses. A repeated measures ANOVA revealed a significant main effect of instruction on self-report ratings of emotion intensity (*F* = 13.04, df = 2, *p* < .001, one-tailed). Pairwise comparisons indicated statistically significant differences between all three levels of self-report ratings in the predicted directions. The Bonferonni corrected *p* value for the criterion of significance for these comparisons was *p* < .016. Self-report ratings were higher following instructions to enhance compared to maintain (mean difference = 0.493, SD = 0.92, *p* = .001; Cohen's *d* = 0.25), and higher following instructions to enhance compared to decrease (mean difference = 0.893, SD = 1.09, *p* < .001; Cohen's *d* = 0.48). They were also higher when asked to maintain compared to decrease initial negative emotional responses (mean difference = 0.399, SD = 0.868, *p* = .004; Cohen's *d* = 0.22).

A manipulation check was conducted before analysing the effects of the emotion regulation instructions on SCR. SCR rate of responses in the 1–4 post-stimulus period (i.e., when participants were presented with the images but had not yet heard the emotion regulation instructions) were not statistically different, as confirmed by a repeated measures ANOVA (*F* = 0.13, df = 2, *p* = .85, two-tailed). The effects of the emotion regulation instructions on SCR regulation values were then analysed knowing that any differences found in SCR following the regulation instruction were produced by the emotion regulation instructions rather than any (presumably random) differences in initial SCR responses to the images.

The SCR regulation values for enhance, maintain and decrease instruction conditions were all negative. This indicated that, across the overall sample, the physiological arousal during the emotion regulation period decreased following each type of instruction. However, as predicted the rate of this decrease was smallest following instructions to enhance (*M* = −0.004, SD = 0.03), greatest following instructions to decrease (*M* = −0.018, SD = 0.03), and in-between following instructions to maintain (*M* = −0.017, SD = 0.03) initial emotional responses (*F* = 6.82, df = 2, *p* < .01, one-tailed). Pairwise comparisons indicated a statistically significant difference between the enhance and maintain instruction conditions (mean difference = 0.013, SD = 0.024, *p* = .002; Cohen's *d* = 0.43), and a statistically significant difference between the enhance and decrease conditions (mean difference = 0.013, SD = 0.027, *p* = .003; Cohen's *d* = 0.46). The Bonferonni corrected *p* value for the criterion of significance for these comparisons was *p* < .016. The difference between the maintain and decrease instruction conditions was not statistically significant (mean difference = 0.001, SD = 0.027, *p* = .832). These findings validated the experimental methodology used in the current study.

### The relationship between emotion regulation and PTSD symptoms

#### Enhancing negative emotions

There was no statistically significant relationship between PTSD symptoms and self-report ratings following instructions to enhance (*r* = −0.003, *N* = 45, *p* = .49, one-tailed). However, there was a statistically significant negative relationship between PTSD symptoms and SCR rate of response when enhancing negative emotions (*r* = −0.37, *N* = 43, *p* = .008, one-tailed). There was no statistically significant relationship between PTSD symptoms and initial SCR values towards unpleasant images (*r* = −0.05, *N* = 43, *p* = .73, two-tailed). Therefore, the relationship reported between PTSD symptoms and difficulty enhancing negative emotions could not be accounted for by individual differences in baseline levels of physiological arousal.

#### Maintaining negative emotions

There was no statistically significant relationship between PTSD symptoms and self-report ratings following instructions to maintain (*r* = 0.096, *N* = 45, *p* = .26, one-tailed). There was also no statistically significant relationship between PTSD symptoms and SCR rate of response after instructions to maintain initial emotional experience (*r* = 0.01, *N* = 43, *p* = .48, one-tailed).

#### Decreasing negative emotions

As in the enhance and maintain conditions, there was no statistically significant relationship between PTSD symptoms and self-report ratings following instructions to decrease (*r* = 0.07, *N* = 45, *p* = .33, one-tailed). There was also no statistically significant relationship between PTSD symptoms and SCR values following instructions to decrease initial emotional responses (*r* = −0.10, *N* = 43, *p* = .26, one-tailed).

### Strategies used to regulate negative emotions on the computer task and PTSD

The descriptions participants gave to describe the strategies they employed to enhance and decrease their negative emotions were categorised according to Gross and Thompson's (2007) model. Inter-rater reliability of these categorisations was 95% across two raters, based on 20% of the data. Conflict was resolved through discussion between the two raters. These data were randomly selected for reliability analysis. The inter-rater reliability suggested a high degree of reliability for the categorisations.

When instructed to enhance negative emotional responses to unpleasant images, 80% (*n* = 36) of participants described engaging in cognitive change (e.g.,“*Tell myself how much worse the situation will become*”), 22% (*n* = 10) of participants reported using attentional deployment (e.g.,“*Looking at more detail of the injuries and situation*”), and 16% (*n* = 7) described employing response modulation (e.g., “*Allowed my emotions to bubble over*”).

When instructed to decrease initial negative emotional responses to unpleasant images, 65% (*n* = 28) of participants described engaging in cognitive change (e.g., “*Looking for the positives in the situation and what could improve*”), 14% (*n* = 6) reported using attentional deployment (e.g., “*Looked at less gruesome bits of the picture*”), 35% (*n* = 15) described employing response modulation (e.g., “*Blanked off*”), and 9% (*n* = 4) described trying an ‘Other’ strategy.

PTSD symptoms were statistically associated with the amount of time spent engaging in cognitive change (e.g. reappraisal) when instructed to decrease negative emotions during the computer task as hypothesised: *r* = −0.25, *N* = 43, *p* = .05 (one-tailed). PTSD symptoms were also statistically related to the amount of time spent engaging in response modulation (e.g. emotional or expressive suppression) when attempting to decrease negative emotions during the computer task, also as hypothesised: *r* = 0.31, *N* = 43 *p* = .02 (one-tailed). The Bonferonni corrected *p* value for the criterion for significance for these comparisons was *p* = .025, indicating trend level significance for the relationship between cognitive reappraisal and PTSD symptoms and statistical significance for the relationship between response modulation and PTSD symptoms. Those with more PTSD symptoms spent less time engaging in cognitive change and more time engaging in response modulation when decreasing negative emotions. However, no statistically significant relationships were found between PTSD symptoms and the amount of time spent engaging in cognitive change (*r* = 0.004, *N* = 45, *p* = .98) or response modulation (*r* = 0.007, *N* = 45, *p* = .96) when participants were required to enhance their negative emotions during the task.

Although not specifically hypothesised, we also explored whether the amount of time spent engaging in emotion regulation strategies was related to SCR when participants attempted to regulate their negative emotions. Self-reported use of response modulation when asked to decrease negative emotions was positively related to higher SCR values (*r* = 0.37, *N* = 43, *p* = .02, two-tailed). Self-reported use of cognitive reappraisal when asked to decrease negative emotions was related to lower SCR values (*r* = −0.30, *N* = 43, *p* = .05, two-tailed). In relation to enhancing negative emotions, there were no relationships between self-reported use of response modulation and SCR (*r* = 0.09, *N* = 43, *p* = .58) and between cognitive reappraisal and SCR (*r* = 0.09, *N* = 43, *p* = .57).

### The relationship between arousal and subsequent intrusive memories

Of the 43 participants whose SCR data were available, 93% (*N* = 40) returned the diary recording intrusive memories for unpleasant images presented during the computer task. Floor effects were evident for some participants with zero intrusions reported for 18 (45%) participants. The range in the frequency of intrusive memories during the week following the computer task was between zero and 13. The median number of intrusions during the week was one and the mean was 2.4 (SD = 3.7). As predicted, there was a significant relationship between decreased arousal during the computer task (measured by SCR) when participants were expected to decrease their arousal (i.e., in response to instructions to decrease their emotions) and the development of intrusive memories in the week following participation, *r* = −0.29, *N* = 40, *p* = .03 (one-tailed). Greater reductions in physiological arousal when instructed to decrease negative emotions were associated with a greater frequency of intrusive memories for images shown during the computer task in the week following participation. As expected, there were no relationships between physiological arousal during the computer task when instructed to enhance (*r* = 0.09, *N* = 40, *p* = .27, one-tailed) or maintain (*r* = −0.11, *N* = 40, *p* = .26, one-tailed) negative emotions and the number of intrusive memories experienced in the week following the computer task.

Since reductions in arousal were associated with subsequent intrusive memories after instructions to decrease initial negative emotional responses, we explored whether initial physiological arousal (i.e. before emotion regulation instructions were given) was associated with subsequent intrusive memories, and thus would explain this relationship. However, correlation analyses revealed no statistically significant relationships between initial SCR values later assigned to enhance (*r* = −0.03, *N* = 40, *p* = .85, two-tailed), maintain (*r* = −0.05, *N* = 40, *p* = .75, two-tailed) and decrease (*r* = 0.14, *N* = 40, *p* = .39, two-tailed) instruction conditions and subsequent intrusive memories. Therefore, the relationship between reduced physiological arousal when decreasing negative emotions during the computer task and the subsequent number of intrusive memories can be considered to be due to participants' attempts to regulate their emotions. There was no relationship between PTSD symptoms and the number of intrusive memories experienced during the week following the computer task (*r* = 0.05, *N* = 40, *p* = .76, two-tailed). Thus, existing PTSD symptoms could not account for the frequency of intrusive memories that followed the computer task.

## Discussion

This novel study investigated the relationship between PTSD symptoms and the ability to regulate negative emotions in real-time whilst measuring SCR and self-report ratings of emotion intensity. It is to our knowledge the first study to explicitly explore the effects of regulating negative emotions on arousal, and the subsequent development of intrusive memories in a sample of trauma-exposed participants.

As predicted, self-report ratings of negative emotion intensity and initial SCR values were greater towards unpleasant images compared to neutral images. Instructions to enhance, maintain and decrease initial negative emotional responses led to changes in the self-report ratings of emotion and SCR values in the predicted directions. These findings validated the methodology used. Greater PTSD symptom severity was associated with lower SCR values after instructions to enhance negative emotions. This finding is suggestive of alternations in emotion regulation associated with PTSD symptoms and is consistent with previous research linking PTSD with difficulty regulating negative emotions (e.g. Bonn-Miller et al., 2011; Cloitre et al., 2005; Eftekhari et al., 2009; Ehring & Quack, 2010; Kashdan et al., 2006; Moore et al., 2008; Price et al., 2006; Tull et al., 2007). However, participants' self-reported emotion when asked to enhance their negative emotions was not associated with PTSD symptoms. This mis-match is consistent with the assertion made by Connelly and Denney (2007) that subjective and objective indicators of emotion regulation do not always correlate. There were no relationships between PTSD symptom severity and difficulties maintaining or decreasing negative emotions when both the SCR values and self-report ratings were analysed.

The relationship between greater PTSD symptoms and difficulty enhancing negative emotions is interesting and warrants careful thought. Since a body of research has demonstrated that healthy participants are able to enhance and decrease negative emotions in response to negative pictures with corresponding effects on physiology (e.g., Kim & Hamann, 2012; Ray et al., 2010), it is possible that our participants with PTSD symptoms experienced genuine difficulty enhancing their negative emotions when required to do so. Our participant sample was comprised of ambulance workers who are likely to be well-practiced in maintaining or down-regulating their emotional responses to traumatic stimuli in their daily work. The results highlighting their difficulty enhancing their negative emotions may reflect the more uncommon task of being required to enhance their emotions when, during their call-outs, they would be expected and practiced at reducing their negative emotion. Alternatively, the results may reflect reduced effort or motivation to enhance negative emotions in those with greater PTSD symptoms. Since emotional avoidance is characteristic of the disorder, it would fit that when negative emotions linked to traumatic stimuli are experienced, there may be attempts to avoid engaging with the negative emotions and hence, difficulties enhancing them. In this way, participants with greater PTSD symptoms may have disengaged with or avoided the task of enhancing their negative emotions when instructed, leading to reduced SCR values. It is also possible that emotional numbing or blocking may have been utilised by those with greater PTSD symptoms. However, analyses indicated that most participants did not engage in response modulation strategies to enhance their negative emotions, and there was no relationship between PTSD symptoms and the amount of time spent engaging in response modulation (which includes emotional numbing and blocking strategies) or in cognitive change strategies to enhance negative emotions. Cognitive change strategies, such as cognitive reappraisal, are effective and commonly used by healthy individuals to enhance negative emotion (i.e., Kim & Hamann, 2012; Ray et al., 2010) and were most commonly reported as strategies adopted to enhance negative emotion during the computer task. It is possible that participants with greater PTSD symptoms attempted but failed to adequately employ cognitive change strategies to enhance their negative emotion when required to do so.

Greater PTSD symptom severity was associated with spending less time engaging in cognitive change (reappraisal) and more time engaging in response modulation (e.g., suppression) when required to decrease initial responses to unpleasant images. This is consistent with research, which shows that response modulation, in particular expressive suppression, is positively related to PTSD (e.g., Moore et al., 2008) and psychological distress following trauma (e.g., Bonanno et al., 2004). The amount of time spent engaging in response modulation strategies was related to greater arousal when participants were asked to decrease their negative emotions. The use of cognitive change strategies, such as cognitive reappraisal, was related to less arousal when decreasing negative emotions. This is consistent with the body of research that suggests that response modulation strategies are inefficient methods for altering physiological responding in the desired directions when attempting to suppress negative emotions (e.g. Gross, 1998b; Gross & Levenson, 1997), and that cognitive reappraisal is more adaptive in reducing physiological responding during attempts to decrease negative emotion (e.g., Gross, 2002). There was no relationship between time engaged in response modulation and cognitive reappraisal strategies and SCR when required to enhance emotions. However, the former finding is likely to be due to floor effects since most participants did not engage in response modulation strategies to enhance negative emotions. Whilst some studies (e.g., Kim & Hamman, 2012; Ray, Ochsner, McRae, & Gross, 2010) have found that cognitive reappraisal strategies are linked to greater physiological responding when enhancing negative emotions, our failure to demonstrate this association may relate to our sample. Since ambulance workers are regularly exposed to traumatic stimuli as part of their job, they may be more adept at decreasing rather than enhancing their negative emotions and as such, physiological responses may be more evident in decrease regulation conditions in this sample. This is consistent with our overall finding that across the sample, physiological arousal during the emotion regulation period decreased following each type of instruction. However, as predicted, the rate of this decrease was smallest following instructions to enhance, greatest following instructions to decrease, and in-between following instructions to maintain initial emotional responses.

Turning to intrusive memories, a greater number of intrusive memories in the week following the task was associated with lower physiological arousal when decreasing negative emotions. This relationship could not be explained by participants' initial arousal before they were instructed to regulate their emotions or current PTSD symptoms. This finding replicates Holmes et al. (2004). Engaging in emotion regulation strategies may disrupt encoding and hence, memory consolidation for the analogue trauma, which may decrease physiological arousal in the short-term but lead to more intrusive memories subsequently (e.g., Ehlers & Clark, 2000). However, since we did not assess the stability of memory for the analogue trauma this is a preliminary hypothesis at this stage. Subsequent intrusions were unrelated to increased physiological arousal when enhancing or maintaining negative emotions, which suggests that it is decreases in physiological arousal, as demonstrated in previous studies, when exposed to analogue trauma that is related to intrusive memory development.

Our results demonstrate that emotion regulation can be assessed via SCR in trauma-exposed individuals with PTSD symptoms. Furthermore, the findings indicate that training in how to regulate emotions is not necessary in such experimental designs. SCR may be a more acceptable mode to objectively measure emotion regulation in this group compared to more invasive and expensive psychophysiological measures such as the assessment of startle eye blink responses, and suggests that this paradigm could be extended to clinical populations. Our results may suggest that difficulty enhancing negative emotions is linked to PTSD symptoms. Since increased PTSD symptoms were related to greater use of response modulation strategies (e.g., suppression) and less use of cognitive change (reappraisal), the results suggest that these symptoms may influence the types of strategies employed to regulate negative emotion. The results add to the growing body of research that points to the adverse consequences and correlates of expressive suppression (e.g., Bonanno et al., 2004; Gross, 1998b; Moore et al., 2008), and underscores the benefits of cognitive reappraisal in effectively modulating emotion. Future research is needed to determine the direction of causality between the use of emotion regulation strategies, such as expressive suppression and the limited use of cognitive reappraisal, and the development of PTSD.

This study has limitations. First, although one third of the sample had PTSD scores in the moderate to severe range, two thirds of the sample had mild levels of PTSD symptoms, which may limit the generalisability of the findings. Future studies should aim to recruit participants with clinical levels of PTSD symptoms so that findings can be considered truly representative of the disorder. Second, ambulance workers are routinely exposed to trauma as part of their job and are likely to utilize emotion regulation strategies that allow them to be effective during emergency situations at work. That is, they are required to be adept at not enhancing their negative emotions in such circumstances. The use of ambulance workers may limit the generalisability of the current findings to other trauma populations. Third, the study may lack ecological validity in terms of the generalisability of the computer task to real-life traumatic events. However, it permitted an objective measure of emotion regulation in real-time. Fourth, the diary used to record intrusive memories perhaps lacked reliability. There was no way of checking that participants completed these accurately. However, this measure has been used frequently in prior research in the field of trauma (e.g., Holmes et al., 2004; Pearson, 2012; Priebe et al., 2013). Finally, the design was not prospective. Subsequent research could employ a prospective design to ascertain the relationship between difficulty regulating emotion and the development of PTSD symptoms.

### Conclusions

This study suggests that emotion regulation can be measured in real-time with a computerised task in trauma-exposed individuals and that physiological measures of arousal may be a more reliable marker of emotion regulation ability compared to self-report. The results suggest that difficulty regulating negative emotions may be a feature of individuals with PTSD symptoms. PTSD symptoms were associated with greater use of response modulation strategies and less use of cognitive change strategies to regulate negative emotions. More intrusions developed in participants who had greater reductions in arousal when attempting to decrease negative emotions.
